# Breast cancer survivorship needs: a qualitative study

**DOI:** 10.1186/s12885-024-11834-5

**Published:** 2024-01-17

**Authors:** Rahimeh Khajoei, Payam Azadeh, Sima ZohariAnboohi, Mahnaz Ilkhani, Fatemah Heshmati Nabavi

**Affiliations:** 1 Student Research Committee, School of Nursing and Midwifery, Shahid Beheshti University of Medical Sciences, Tehran, IR Iran; 2 Radiation Oncology Department, School of Medicine, Imam Hossein Hospital, Shahid Beheshti University of Medical Sciences, Tehran, Iran; 3 Department of Medical Surgical Nursing, School of Nursing and Midwifery, Shahid Beheshti University of Medical Sciences, Tehran, IR Iran; 4 Nursing and Midwifery Care Research Center, Mashhad University of Medical Sciences, Mashhad, Iran

**Keywords:** Breast cancer, Survivorship, Needs

## Abstract

**Background:**

Breast cancer rates and the number of breast cancer survivors have been increasing among women in Iran. Effective responses from healthcare depend on appropriately identifying survivors’ needs. This study investigated the experience and needs of breast cancer survivors in different dimensions.

**Methods:**

In this qualitative content analysis, semi-structured in-depth interviews were conducted from April 2023 to July 2023. Data saturation was achieved after interviewing 16 breast cancer survivors (BCSs) and four oncologists using purposive sampling. Survivors were asked to narrate their experiences about their needs during the survivorship. Data were analyzed with an inductive approach in order to extract the themes.

**Results:**

Twenty interviews were conducted. The analysis focused on four central themes: (1) financial toxicity (healthcare costs, unplanned retirement, and insurance coverage of services); (2) family support (emotional support, Physical support); (3) informational needs (management of side effects, management of uncertainty, and balanced diet); and (4) psychological and physical issues (pain, fatigue, hot flashes, and fear of cancer recurrence).

**Conclusions:**

This study provides valuable information for designing survivorship care plans. Identifying the survivorship needs of breast cancer survivors is the first and most important step, leading to optimal healthcare delivery and improving quality of life. It is recommended to check the financial capability of patients and take necessary measures for patients with financial problems. Additionally, support sources should be assessed and appropriate. Psychological interventions should be considered for patients without a support source. Consultation groups can be used to meet the information needs of patients. For patients with physical problems, self-care recommendations may also be useful in addition to doctors’ orders.

## Introduction

According to the Globocan website, breast cancer is the most common cancer among women. Breast cancer was the most common cancer in Iran in 2020 in both sexes and the fifth cause of death in both sexes; however, it is the leading cause of death in women [1]. Due to early diagnosis and improvements in treatment methods, the prognosis and survival rate of women with breast cancer have enhanced significantly worldwide [2]. Breast cancer survivors face many problems and needs in different dimensions: psychological, physical, social, etc. [3]. These needs and issues during the survivorship period are different during the active phase of cancer treatment [4]. Physical and psychological symptoms include fatigue, pain, osteoporosis, premature menopause, fear of disease recurrence, sexual problems, and infertility [5–7]. To deal with these health needs, many patients require medical, psychological, and social care for more than ten years after diagnosis [8, 9].

The growing population of survivors, along with their multiple needs, is a challenge for healthcare providers and health policymakers, who need to provide a good standard of care during the survival period and meet the different dimensions of the needs of these patients after treatment [3, 10]. Failure to meet these needs is associated with negative consequences such as decreased satisfaction with care, poor adherence to treatment, decreased quality of life, and increased anxiety and depression [11]. The current trend in modern medicine is changing from a disease-based model to a patient-centered model in which patients are active and their preferences and needs are considered in care [12]. Therefore, the first step in planning supportive care services for cancer patients is to identify their care needs [13]. To date, several studies have investigated the needs and experiences of breast cancer survivors and obtained different results. The need for help in dealing with problems such as financial distress [14], Pain [15], Memory problems [16], Fear of disclosure [17], concerns relating to body image, femininity, altered physical appearance, and self-confidence [18] are among the needs experienced by breast cancer survivors. In 2023, a systematic study on the needs of breast cancer survivors showed that most of these patients had psychological and informational needs [19]. In assessing the needs of BCSs, it is essential to consider culture, ethnicity, socioeconomic status, birthplace, and native language. The elements of the survivorship journey may be somewhat similar for all women, yet we cannot assume that their experiences are monolithic, regardless of the context. Ethnic, racial, and cultural diversity may affect survivors’ needs. Iran is a country with unique cultural and religious characteristics and people’s behavior and attitude towards disease are different depending on their culture. As such, conducting research across cultures is necessary. For example, the family holds a valuable position in Iran. Cancer affects physical, mental, social, and economic dimensions of patients and their families. In this situation, preserving and consolidating the family foundation is important and may create different needs for patients. Therefore, considering the differences in the ethnic, cultural, and socioeconomic background of Iran, this study aimed to investigate the needs and experiences of breast cancer survivors in the country.

## Method

This qualitative study was conducted using a conventional content analysis approach in order to investigate the experiences and needs of breast cancer survivors. Qualitative research methods seek to discover and understand people’s inner worlds. As experiences form the structure of truth for each person, researchers can discover the meaning of phenomena from their perspective by taking into account people’s experiences [20]. In conventional content analysis, classes are extracted directly from the data text. This approach is used in studies whose purpose is to describe a phenomenon; this method was used in the present study [21].

## Contributors

The qualitative research participants had deep experience of the phenomenon under study [19]. The participants in the study were breast cancer survivors (14 participants), patients’ families (two participants), and healthcare professionals (four participants). Women who had completed their treatment courses at a university hospital in Tehran between four and 18 months prior and were visiting the hospital for follow-up care were included in the study using targeted sampling. To achieve the maximum difference in the samples, participants with maximum diversity were included in the study over a period of three months. Participants of different ages, marriages, education levels, income, and ethnicities were included in the study. The sampling was continued until data saturation was achieved. The inclusion criteria were patients between 18 and 60 years of age, literacy, completion of treatment, non-metastatic cancer, and stable clinical conditions. The exclusion criteria were cancer recurrence or metastasis, secondary cancers, and cognitive impairment.

### Collecting data

Two separate interview guides for survivors and healthcare professionals were developed by the research team, using expert input on the needs of cancer survivors. These guidelines are based on a literature review of the needs of survivors of breast cancer. In-depth semi-structured interviews were conducted in order to collect the data. After informed consent was obtained, an interview method with open-ended questions focusing on the study objectives was conducted. Each interview was conducted and analyzed. The interviews were scheduled at a convenient time and location for the interviewees. A total of 20 face-to-face and telephone interviews were conducted, lasting 45 min each. The data were collected between April 20 and July 30, 2023. The interviews began with an open-ended question relating to the needs and experiences of breast cancer survivors. Based on the interviewees’ answers, the interview process was directed towards achieving the main goal of the research. Exploratory questions were asked in order to attain a deeper understanding of the phenomena. The interviews continued until complete information saturation was reached. Full saturation occurs when classes and subclasses are completed, and new data do not add anything to these classes; at this point, no new data are obtained from the interviews and only previous information is repeated. Immediately after the end of the interviews, all interviews were recorded verbatim using the recording device and transcribed.

The interview guide questions included the following questions:

-  What were the most important issues you faced after completing your treatment?
-  What side effects did you experience and how did you deal with them?
-  What feelings did you experience during your recovery? Fear, anxiety, depression, despair, anger, fear of cancer returning, loss of self-confidence?
-  Explain your emotional life.

In addition, according to the participants’ answers, the following questions were addressed:

-  In what areas did you need more training and information?
-  What physical problems and complications do you currently face in need of support?
-  Do you need support in establishing relationships with others and being present in the community and workplace?
-  Do you feel that you need more support in your emotional and family relationships?
-  Do you need help from others in performing daily tasks?
-  Do you have trouble remembering and recalling events?

In addition, interview guide questions for healthcare providers include the following:

-  From your perspective, what are the most important issues and needs of breast cancer survivors? (in physical, mental, informational, cognitive dimensions, etc.)
-  Are there any issues that are more problematic for the Iranian recoveries?

The researcher further explored participants’ answers to each question. Furthermore, by restating and returning to the salient points or a summary of the participants’ answers, we confirmed the correctness of the data and increased the credibility of the results. At the end of each session, we asked questions such as “Is there anything else you would like to add?” and “Is there another question I should have asked?” Participants were asked to express any further experience or additional information. In addition, permission was obtained from the participants for future calls or interviews. At the end of each interview, the interviews were listened to several times at the earliest possible time and transcribed verbatim. The moods and characteristics of the participants were recorded along with the interviews. The mood and characteristics of the participants helped code the text of the interviews. That the codes accurately express a person’s understanding of the desired situation. The text of the interviews with pseudonyms was entered into MAXQDA 2020 software for data storage, retrieval, and analysis.

### Data analysis

Data analysis was performed using conventional content analysis, based on the steps introduced by Graneheim and Lundman [22]. The sentences and phrases describing the needs and experiences of survivors were coded. The first and third researchers coded the texts of the interviews separately. The coded text was reviewed by a responsible researcher. Any disagreements were discussed to reach a consensus. Subsequently, the central concept of each class and the main and abstract concepts were defined. Following this, based on a constant comparison of similarities and differences, the codes that indicated a single topic were placed in a class, the subclasses and classes were categorized, and the core codes were formed. In the fifth step, the central concept of each class and the main and abstract concepts were defined.

### Data integrity and robustness

To validate the research findings, the criteria of acceptability or validity, transferability, reliability, trust or stability, and ability, based on Lincoln and Guba, were used [23]. To ensure the validity of the data, in addition to allocating sufficient time for data collection and immersion, semi-structured interviews were conducted; the maximum variety in sampling was observed by interviewing people of different ages, education, income, and use at various stages of the disease. Sampling continued until the data reached saturation and the most appropriate semantic unit was selected. Internal validity of content analysis was condcuted; this was evaluated for its face validity. To validate the content, a panel of experts (research team) supported the generation of concepts or coding topics; these were also reviewed by the participants. For this purpose, the interview text and extracted codes were presented to participants, who commented on their accuracy [24]. In this study, a research audit, a detailed review of data by an external observer, was used to increase the stability of the research. In addition, the period of data collection (interviews) was carried out as quickly as possible and all participants were asked about the same topic [22]. To facilitate transferability, the researcher has provided a clear description of the platform, the method of selecting and characterizing the participants, the data collection, and the analysis process so that the reader can judge the applicability of the findings to other situations. In addition, rich and detailed findings with appropriate quotations and authentic documents are presented in order to increase transferability [25]. To increase the verifiability of the data, all research stages, methodology, and decisions taken in the research stages are explained in clear detail so that they can be followed by other researchers if necessary. In addition, all raw data and recorded notes, documents, and interviews have been retained for future review [26].

### Ethical considerations

This study was approved by the Research Ethics Committee of Shahid Beheshti University of Medical Sciences in Tehran (IR. SBMU. PHARMACY. REC.1402.005). Informed consent was obtained from the research participants, and they were also informed about the confidentiality of their information.

## Results

### Participants

The medical records of 450 BCSs were reviewed. 56 patients met the inclusion criteria for this study. 28 survivors could not be contacted, 12 declined to participate, and 16 participated in the interviews. Among the survivors, 64% were married and about half were between 30 and 50 years old. 60% had a higher education level and over 85% had an average income. Of the 12 oncologists who were invited to participate in this study, four agreed to participate, with a response rate of 33.3%.

### Themes

The analysis focused on four central themes: (1) financial toxicity (healthcare costs, unplanned retirement, and insurance coverage of services); (2) family support (emotional support, Physical support); (3) informational needs (management of side effects, management of uncertainty, and balanced diet); and (4) psychological and physical issues (pain, fatigue, hot flashes, and fear of cancer recurrence).

#### Financial toxicity

The high cost of drugs and treatment, along with reduced work productivity and subsequent loss of income, impose a heavy financial burden on cancer patients, which in turn imposes a unique stress known as financial toxicity [27]. In this study, most patients mentioned financial problems as their most challenging issue.

##### Healthcare costs

One source of financial problems is the costs associated with cancer care services (e.g., medications, supplies, copayments, and transportation) [28]. Patients who report cancer-related financial problems or high healthcare costs are more likely to avoid or delay their medical care or prescribed medications. This may slow their healing process or aggravate the disease [29]. The participants in this study complained about the high costs of drugs and medical procedures. One participant said:

I had finished chemotherapy three months ago and was told to start radiation. But I couldn’t afford it, so I gave it up.

##### Coverage of insurance services

Another financial problem for the participants was that insurance did not cover the cost of some treatments. Obtaining health insurance did not fully protect against cancer-related financial problems. One participant said:

I am a worker and I have to work hard to improve my wife’s condition. Thank God, most of my wife’s treatment costs were paid by supplementary insurance. However, now the problem is that my wife has to undergo a mastectomy and she is very upset about the change in her appearance and wants to have breast implants. However, because it is considered a cosmetic procedure, unfortunately, insurance does not accept the payment and we are not able to pay the cost.

##### Unplanned retirement

One source of financial distress is reduced income due to loss of employment, missing work, or unplanned retirement [28]. In this study, it was found that some patients were forced to retire and had difficulty with paying the treatment costs. One participant said:

I am a teacher, and I was forced to retire due to illness. My pension is not sufficient to pay for my medicine and treatment. I really don’t know what to do to pay for the treatment.

#### Family support

##### Emotional support

A remarkable finding obtained from the participants’ statements was that emotional support from their family, partner, and children played an important role in reducing their physical and psychological symptoms. Survivors who had a strong source of emotional support experienced fewer physical symptoms and a better mental state; among these patients, psychological symptoms such as stress, anxiety, and depression were far less common. Studies have shown that BCSs face various stresses during the course of the disease and need comprehensive support, including social and family support, to deal with these stressful factors and manage their resulting conditions better [30]. Family involvement increases their ability to cope [31]. Some of the participants’ statements support this:

After the radiation treatment, I felt much better because I went to my family in the city. They supported me a lot, making me feel better.

I live alone. Initially, my family supported me, but they left me little by little. Of course, they are right, our problem is ongoing, and I do not like to disturb anyone. I am struggling with several complications. The current problems include severe muscle pain, fatigue, weakness, and lethargy. I feel lonely. I often feel stressed and anxious.

The husband of one of the participants said:

My wife is not experiencing any particular problem at the moment because I am paying attention to her in every way. She is my life partner, and I should be by her side facing difficulties. I will do whatever I can to make her feel better. Thank God; there is no problem now.

Considering that partners play an essential role in providing emotional support, financial management, and decision-making regarding their spouses’ cancer treatment, there is a need to involve them in psychological interventions [32].

##### Physical support

Due to the complications of the treatment, the participants needed physical support from the family to fulfill their responsibilities. Patients who lacked physical support from the family had many problems in carrying out daily responsibilities and activities. One participant said:

I separated from my wife and live alone. My hand is swollen and painful, but I have to work with this condition; And I don’t have anyone to help me and this is really hard for me.

#### Informational needs

Almost all participants stated that they did not receive any information on what to expect. and would have liked to receive more information about management of side effects and balanced diet.

##### Management of side effects

Information needs were one of the most important needs for the survivors. Most participants said that they had not been given any training program. Many women reported that they had experienced persistent side effects from treatment for which they were poorly prepared. Moreover, they did not receive any related training. This lack of information confused them and exacerbated complications. One participant said:

I was not provided with any training. I was faced with cases where I did not know what to do. For example, because of menopause, I used to get hot flashes and I was very bothered. Later, they said you could use cool liquids like chicory, but I did not know what to do, and I tolerated it.

##### Management of uncertainty

One of the informational needs for BCSs was ensuring complete recovery. They were confused about being completely healed. Furthermore, they wanted more information about disease prevention for their daughters. One participant said:

My most important question during the recovery period is whether I have fully recovered. Does cancer have a chance of coming back? Is it possible for my daughter to develop cancer in the future?

One of the specialists stated:

Facing an uncertain future and the fear of the disease returning (especially the concern for children) is one of the most important concerns of BCSs. They should know that creating a suitable lifestyle and continuous follow-up can prevent the return of the disease.

##### Balanced diet

Another need expressed by patients was information about healthy eating or balanced eating (food to eat, food to avoid). One of the participants said:

In this regard, we did not receive sufficient training. I do not know what food is good for me. There is some information on the internet, but it is unreliable. I cannot always ask my doctor these questions. They should teach us about proper nutrition.

#### Psychological and physical issues

##### Pain

Most participants reported post-mastectomy, chest/arm, musculoskeletal, and lymphatic pain. Chronic pain is one of the most troublesome side effects of breast cancer treatment and affects patients’ quality of life [33]. Chronic pain causes discomfort and fatigue, reduces appetite and sleep, and interferes with many activities of daily living [34]. Some of the participants’ statements regarding pain are provided below. One of the specialists stated:

Survivors face long-term complications of treatment, such as myalgia, osteoporosis, and hot flashes due to hormonal treatments, respiratory disorders due to receiving radiotherapy, and paresthesias of limbs due to receiving chemotherapy drugs, and they should have the necessary information and background to face these complications.

Participants stated:

My main problem was that I experienced so much musculoskeletal pain that I could not move. I was admitted to the hospital a couple of times, and a bunch of drugs were injected into me, but it worked temporarily, and the pain recurred.

My left hand is in severe pain due to the removal of the lymph nodes, which makes it difficult for me to do my chores and I need the help of others.

##### Fatigue

Fatigue was another physical symptom mentioned by most participants. Fatigue is the most common and frequent symptom of BCSs after treatment [35]. Studies have shown that low physical activity, decreased muscle mass, and impaired physical fitness are potential causes of fatigue in BCSs [36]. One participant said:

Sometimes I am so tired that I have to take a break several times while doing a normal daily task and continue again.

##### Hot flashes

Most of the participants stated that they experienced hot flashes due to menopause. Hot flashes are a common problem in breast cancer survivors, affecting their work performance [37]. One of the participants stated:

I have night sweats and hot flashes due to menopause. It bothers me a lot; it starts with my head and then with the rest of my body. Anyone around me will notice that I am sweating profusely. When I get this condition at night, I wake up, get annoyed, and cannot sleep anymore. I drink cold liquids, and it gets better but not completely.

##### Fear of cancer recurrence

Fear of cancer recurrence is one of the most commonly reported concerns among cancer survivors [38]. Most participants experienced FCR; it was the most important source of stress and worry after breast cancer diagnosis and treatment, involving the fear of cancer returning to the breast, metastasizing, or returning as a second cancer. One of the contributors said:

I do not have much stress and anxiety because my doctor told me that my disease has progressed. My only concern is whether my cancer returns?

## Discussion

To the best of our knowledge, this is the first study to examine the needs and experiences of Iranian BCSs. The most important finding of this study was the patients’ experience with financial problems. Most patients had financial problems associated with cancer care services (e.g., therapeutic procedures, supplies, medications, and transportation) and reduced income due to loss of employment, missing work, or unplanned retirement. In Iranian government hospitals, all healthcare services are based on insurance. Patients who use these services in hospitals are required to pay a percentage of the treatment cost. Usually, cosmetic procedures are not included in these insurance programs. However, the increase in inflation and prices has caused most patients to experience financial problems. This finding is consistent with that of a study by Autade and Chauhan, in which all BCSs reported financial problems [39]. In addition, in a study by Carrera (2018), 28–48% of cancer survivors experienced financial toxicity based on monetary measures and 16–73% experienced financial toxicity based on subjective measures [40]. In a study by Barthakur et al. (2016), finances relating to treatment were a major concern for survivors and there was a need for information on reducing healthcare costs [41]. Over 8% reported that providing for the financial needs of their families was a severe problem [42]. Patients who report cancer-related financial problems or high costs may be more likely to forgo or delay prescription medications or medical care [29]. The inability to afford household expenses is one of the most commonly reported reasons for delayed medical care among cancer patients [43]. Financial problems, if not identified, can negatively affect the physical, psychological, and socioeconomic status of survivors and lead to poorer access to health services and consequently poorer health status and health-related quality of life [44]. Research shows that health professionals recognize patients’ financial concerns but may not be qualified to address them [44]. Healthcare providers can take several steps to reduce patients’ financial difficulties, including: (1) considering the cost of multiple treatment regimens with similar effects, (2) providing cancer cost estimates to patients, (3) considering treatment benefits, (4) assessing patients for financial toxicity, and (5) educating and assisting patients about insurance benefits and other financial assistance institutions that may be available to them [42].

Another finding of this study relates to patients’ experience of support resources. Consistent with Iranian culture, in this study family members were a valuable and main source of support for the BCSs. In the study of Lee et al., family members, including adult children and spouses, were the main source of support for all participants [45].

Upon receiving emotional support from their family, patients felt reassurance, acceptance, and attention. Those who had family support felt less anxious and depressed, and their physical symptoms were much fewer. The effective role of emotional support for patients has been reported in several studies [5, 9, 46]. Wilson et al. reported that support from friends and family is a positive strategy for reducing stress and anxiety and coping with illness [47]. In a study by Moradi et al. (2013), supportive care needs in women with breast cancer decreased after their husbands were educated about familiarity with breast cancer disease, symptoms and complications of the disease, and treatment. It has been suggested that caregivers use training sessions to reduce the needs of women with cancer [48]. These results were inconsistent, however, with those of Thewes (2004) and Arora (2007) [9, 49]. They both concluded that friends and family members of patients with cancer tend to decrease or withdraw their support after treatment completion. The family’s role in improving patient outcomes cannot be overemphasized.

The study shows that patients who do not have access to a family support source have a greater tendency to use social support networks [50]. The researchers could create access to social support networks for patients who do not receive family support. Additionally, the role of support groups cannot be ignored. Support groups consisted of people with common experiences. In Iran, there are many support groups for cancer patients. Experiencers with any type of cancer can declare their readiness to participate in these groups by registering on related sites. These groups usually have weekly or monthly meetings. These meetings are held in person or virtually, according to the position of the group members. According to the needs of the members, the content of these meetings can be informative, reminiscent, fun, sports, cooking, or any topic desired by the majority of the members. Chou et al. (2016) showed that support groups help reduce psychological distress and increase the quality of life of patients with breast cancer. It is also possible to educate patients’ families about the needs of those who have recovered. Moghaddam Tabrizi et al. (2018) conducted an interventional study and trained patients’ families in family participation, optimism, coping with cancer, reducing uncertainty, and managing symptoms. The findings indicated a significant improvement in overall cancer coping scores on all subscales, including individual, positive focus, coping, deviation, planning, and interpersonal communication [31].

The participants also stated that they had unmet information needs in the areas of management of side effects, nutrition, and uncertainty management. Previous studies have also identified informational needs as the most common need for BCSs [3, 5, 51], even for survivors who have been diagnosed and treated for several years. These needs often include information on diet and nutrition, mental health counselling, infertility, and spiritual counselling [52]. Previous studies have indicated that BCSs often feel unready for the side effects that linger after therapy [17, 18]. In a study by Pembroke et al., almost half of the participants reported informational needs associated with side effects of treatment [52]. In this study, patients met their information needs through the Internet; however, they had doubts regarding the use of this information. The high use of the Internet by patients with cancer suggests that healthcare providers may not be adequately meeting patients’ information needs [53].

Germino et al. (2013) conducted an intervention study with the aim of determining the impact of uncertainty management intervention on reducing uncertainty, better uncertainty management, fewer breast cancer-specific concerns and more positive psychological outcomes. The intervention consisted of a written CD and a manual with four weekly 20-minute training calls. The findings showed that BCS who received the intervention reported a reduction in uncertainty and significant improvement in behavioral and cognitive coping strategies for managing uncertainty, self-efficacy, and sexual dysfunction [54].

Physical problems were also reported by most participants. The patients mostly complained of pain, fatigue, and hot flashes. This finding is consistent with the results of the present study [9, 16, 17, 39]. In the study by Bu et al. (2022), fatigue and pain were reported in 40.7% and 37.2% of the subjects respectively [55]. Studies have shown that the use of exercise-based interventions plays an effective role in reducing the pain and fatigue of breast cancer survivors [56, 57].

Studies have also shown that mobile health education intervention improves cancer-related fatigue among breast cancer survivors [58]. mHealth interventions involve the adoption of mobile technologies to provide educational information, help users manage their own conditions and behaviors, and deliver healthcare to improve the health of users [59]. Integrating training to manage fatigue as part of routine care among breast cancer survivors is recommended [58].

The most important mental disorder experienced by patients was the fear of recurrence. The results of this study are similar to those of a previous study, which showed that the most common unmet need was help with coping with the fear of recurrence [60–62]. In the study of Brennan et al. (2010), fear of cancer recurrence was reported by 38% of the participants and emerged as the highest-ranked unmet need in all age groups [63]. In this study, fear of recurrence was identified as a key issue for cancer survivors [64–67].

The remarkable findings obtained from the interviews with health care professionals were that they mentioned the long-term complications caused by treatment procedures such as radiotherapy and chemotherapy in breast cancer survivors, including pain, fatigue, and hot flashes. Survivors have also reported these complications. According to healthcare professionals, facing an uncertain future, fear of cancer recurrence, and body image concerns are the most important concerns for breast cancer survivors. These results are consistent with those reported by Wells et al. [68].

### Limitations

This study has several limitations. First, while the BCSs were diverse, all were recruited from a comprehensive cancer center in Tehran, where they were referred to receive survivorship care. They may differ in other centers. Second, we did not use random sampling to obtain a sample of study participants. Instead, we used purposive sampling, a sampling approach commonly used in qualitative research, in order to select participants who were particularly knowledgeable about the phenomenon under study. Third, a qualitative method was used in this study, which does not allow the testing of a specific hypothesis. Fourth, we used literate participants; different results may be obtained from illiterate individuals.

## Conclusion

The results of this study can be used to design survival care programs for Iranian BCS. Therefore, patients’ support resources should be reviewed and measured. Appropriate psychological interventions could be used for patients who do not have a source of support. The financial ability of patients should be assessed, and necessary measures should be considered for patients with financial problems. All the patients were provided with an educational program tailored to their needs. Consultation groups could be used to meet the information needs of the patients. In patients with physical problems, self-care recommendations may also be useful in addition to doctors’ orders.

## Data Availability

The datasets analysed during the current study are not publicly available due to ethical restrictions. but are available from the corresponding author on reasonable request.

## Abbreviations

BCS: Breast Cancer Survivor
SCP: Survivorship care plans
